# Validation of red blood cell flux and velocity estimations based on optical coherence tomography intensity fluctuations

**DOI:** 10.1038/s41598-020-76774-z

**Published:** 2020-11-11

**Authors:** Paul J. Marchand, Xuecong Lu, Cong Zhang, Frédéric Lesage

**Affiliations:** 1 Department of Electrical Engineering, Polytechnique Montreal, Montreal, Canada; 2 Research Center, Montreal Heart Institute, Montreal, Canada

**Keywords:** Neuro-vascular interactions, Multiphoton microscopy, Interference microscopy, Imaging and sensing, Blood flow

## Abstract

We present a validation of red blood cell flux and speed measurements based on the passage of erythrocytes through the OCT’s focal volume. We compare the performance of the so-called RBC-passage OCT technique to co-localized and simultaneously acquired two-photon excitation fluorescence microscopy (TPEF) measurements. Using concurrent multi-modal imaging, we show that fluctuations in the OCT signal display highly similar features to TPEF time traces. Furthermore, we demonstrate an overall difference in RBC flux and speed of 2.5 ± 3.27 RBC/s and 0.12 ± 0.67 mm/s (mean ± S.D.), compared to TPEF. The analysis also revealed that the OCT RBC flux estimation is most accurate between 20 RBC/s to 60 RBC/s, and is severely underestimated at fluxes beyond 80 RBC/s. Lastly, our analysis shows that the RBC speed estimations increase in accuracy as the speed decreases, reaching a difference of 0.16 ± 0.25 mm/s within the 0–0.5 mm/s speed range.

## Introduction

The microvascular bed plays a crucial role in cerebrovascular physiology, as it is the main site of exchange of oxygen and nutrients to cerebral tissue. Nevertheless, quantitative hemodynamic imaging of the microvascular network remains particularly challenging, due to the small size of capillaries (> 8 µm), single-file nature of their flow and their highly heterogeneous hemodynamic properties and geometries. Over the past decades, two-photon excitation fluorescence (TPEF) microscopy has become the mainstay for quantitative microvascular imaging, through its high resolution and high sensitivity^1^. However, its limited field-of-view hampers its ability to capture microvascular dynamics over large capillary networks at high acquisition speeds. In these regards, optical coherence tomography^2^ (OCT) is an interesting and promising candidate, through its minimal-invasiveness, fast volumetric acquisition speeds and large attainable imaging depths.

Throughout the past decade, a plethora of OCT techniques have been developed to capture hemodynamic parameters such as blood hematocrit, flow and velocity. Doppler OCT^3–5^, a phase-resolved OCT technique, measures the Doppler shift experienced by light back-scattered from moving red blood cells (RBCs) to capture the axial velocity component of RBCs. Since its inception, extensions of Doppler OCT have been developed, to improve its accuracy^6,7^, measure the RBC’s total velocity^8–10^ or alleviate the impact of discrete RBC flow in capillaries on velocity estimations^11,12^. In parallel to these efforts, methods analyzing the OCT signal’s temporal decorrelation have been developed to estimate the velocity of RBCs in large vessels and capillaries^13–15^, and were employed to capture the cortical laminar response to sensory stimulation^16^. In addition to measuring blood flow velocity, a number of OCT techniques have been developed to estimate red blood cell concentration or flux (RBC/s), based primarily on computing the spectral power of the dynamic component of the OCT signal, as in optical coherence micro angiography (OMAG)^17–20^. Various derivations have shown either linear or sublinear relationships between the OCT signal and scatterer concentration, depending on the normalization or sampling time. Lastly, a group of techniques, termed RBC-passage^21^ or particle-counting OCT^22^, have been developed to estimate RBC flux and speed based on the presence of peaks in the OCT signal in capillaries, assumed to originate from the passage of single RBCs in the focal volume. These techniques estimate the RBC flux and speed by counting the number of peaks (or particles) in a voxel time trace and estimating their temporal spread respectively but questions remain about aliasing artefacts and its quantitative potential. Altogether, despite the important number of OCT techniques listed above, most were validated in highly controllable imaging conditions, using intralipid or blood solutions flowing through microchannels. Seldom validations have been performed in vivo, and were compared either against non-validated metrics^12,18^, or sequentially, using two separate imaging setups^14,23^.

In this manuscript, we present a validation of the RBC-passage OCT technique with TPEF microscopy, the current gold-standard for microscopic cerebral hemodynamic imaging. In order to underpin the origins of the fluctuations observed in the OCT signal and assess their accuracy in estimating RBC flux and speed, we constructed a multimodal imaging platform, enabling simultaneous imaging of single capillaries with OCT and TPEF microscopy. Ultimately, we quantify the performance of the RBC-passage technique in measuring both the RBC flux and speed, and highlight its limitations and potential improvements.

## Results

### Evaluation of RBC flux using OCT

**Figure 1:**
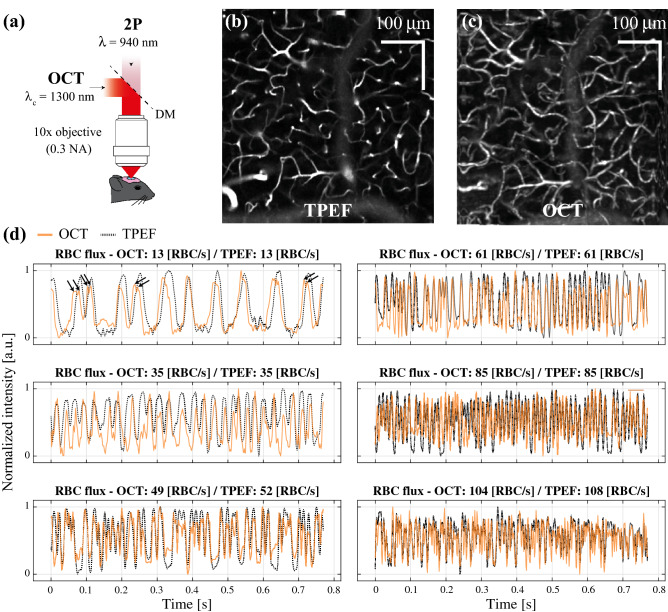
Simultaneous and co-localized OCT and TPEF imaging. (**a**) Optical setup combining OCT and TPEF microscopy through the same objective. (**b**–**c**) angiograms obtained with TPEF and OCT at a depth of 100 µm under the pia. (**d**) examples of time traces acquired in OCT (orange) and TPEF (black dotted line) for various RBC fluxes.

To evaluate the performance of an OCT technique based on measuring the peaks in intensity caused by the passage of RBCs in the focus, we developed a multimodal system combining OCT and TPEF microscopy (Fig. 1a) and measured the hemodynamic activity over n = 119 vessels for an average of ~ 60 s each, simultaneously with both modalities. Although the nature of the contrast differs between OCT and TPEF, our system enables imaging similar vascular structures simultaneously, as illustrated in Fig. 1b and c. Due to the small NA of the objective (NA = 0.3), we limited our measurements to two depths, z = 50 and 100 µm below the pia. Although imaging below these depths would have been possible, the SNR of TPEF images would have likely made the RBC flux and speed estimations inaccurate. The OCT technique used here to quantify RBC flux and speed relies on a temporary increase in back-scattering caused by the passage of a red blood cell in the OCT’s focus. Such an increase in back-scattering will reflect itself in the OCT time traces as a temporary fluctuations in overall magnitude of the OCT signal. As is illustrated in Fig. 1d, the fluctuations in some OCT intensity time traces show slightly different magnitudes, timings and widths, however the overall timeseries highly resemble those obtained in TPEF. Interestingly, for low RBC fluxes (< 20 RBC/s), the OCT traces display some additional fluctuations, either during the passage of the RBC or between RBCs. For very high RBC fluxes (> 35 RBC/s), the OCT time trace oscillates spuriously but still seems to overall follow the TPEF timeseries (in particular by identifying RBC free regions).

**Figure 2:**
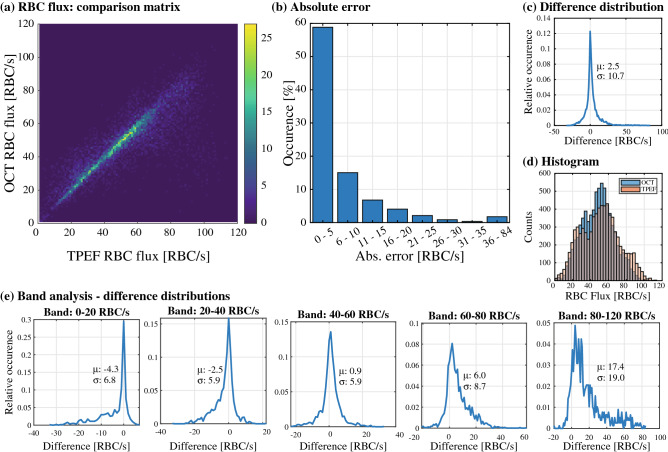
Evaluation of OCT RBC flux estimation. (**a**) 2D histogram displaying the estimation of RBC flux in both modalities, highlighting a concentration of measurements on the diagonal. (**b**) Absolute error between both modalities, binned into steps of 5 RBC/s. (**c**) Distribution of the differences in estimation for all measurements, with a mean of 2.5 RBC/s and variance of 10.7 RBC/s. (**d**) Marginal histograms of OCT and TPEF RBC flux estimations, highlighting a bias in OCT RBC flux towards the 20–60 RBC/s band. (**e**) Band analysis displaying the difference distributions for 5 bands of RBC flux: 0–20, 20–40, 40–60, 80–120 RBC/s.

As described in the methods section, to evaluate the performance of RBC flux measurements using the OCT time traces, we analyzed n = 119 vessels (48, 59 and 12 for each mouse respectively). For each vessel, we extracted the RBC flux in the OCT and TPEF time traces for blocks of 1 s, producing n = 7522 observations. A histogram comparing each RBC flux measurement obtained with both OCT and TPEF is shown in Fig. 2a. The 2D histogram displays an overall linear relationship between the two metrics (slope of a = 0.91, see Supp. Figure 3) as a large concentration of the points lies on its diagonal. The absolute error between both metrics, defined as the absolute difference between the fluxes obtained in OCT and in TPEF, lies in majority between 0 and 5 RBC/s as shown in Fig. 2b. Larger differences are decreasingly frequent, but errors up to 84 RBC/s are nevertheless observed. The overall difference distribution for all bands, shown in Fig. 2c, is symmetrical, has an average value of 2.5 RBC/s and a variance of 10.7 RBC/s (difference defined as Δflux = flux_TPEF_ – flux_OCT_). Interestingly, the width of the 2D histogram’s diagonal (displayed in Fig. 2a) seems constant up to 60 RBC/s and increases for larger fluxes. This observation is clearer when comparing the marginal histograms for OCT and TPEF (Fig. 2d), as the OCT measurements displays more observations within the middle bands (20–60 RBC/s) than in the tails (0–20 RBC/s and 60–100 RBC/s) compared to the TPEF measurements. This is further confirmed in a sub-band analysis, wherein we computed the difference distributions for 5 sub-bands (0–20 RBC/s, 20–40 RBC/s, 40–60 RBC/s, 60–80 RBC/s and 80–120 RBC/s). As can be seen in Fig. 2e, the middle bands (i.e. 20–60 RBC/s) show the smallest average difference and variance. Although the difference is slightly worse for the lower band (large increase in average value but slight change in variance), it is increasingly worse for large values of RBC flux.

**Figure 3:**
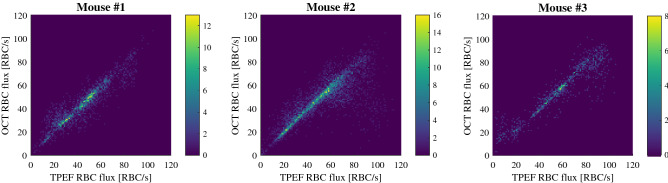
2D histograms highlighting the performance of RBC flux estimation between animals.

The performance of the RBC metric is highly repeatable between animals, as can be seen in the 2D histograms displayed in Fig. 3. Similarly to the overall distribution, the distribution is linear with a slight underestimation in the OCT metric (slopes of 0.96, 0.89 and 0.94 for the three mice respectively). Interestingly, mouse #2 displays a concentration of underestimated points (lower left quadrant of the 2D histogram), originating from the same vessel, which might explain the lower slope observed for this mouse.

### Evaluation of RBC speed using OCT

In addition to assessing the performance of the technique for measuring RBC fluxes, we compared the RBC speed estimation obtained in OCT and in TPEF. As described in the Methods and in Lee et al.^21^ and Ren et al.^22^, the speed in OCT is hypothesized to be estimated by measuring the width of each peak and converting to velocity by assuming the RBC width. In contrast to the RBC flux measurement, the linescans and OCT traces’ length were maintained at 512 time points, and poor TPEF speed estimates were removed from the analysis, thus producing n = 6673 velocity observations.

**Figure 4:**
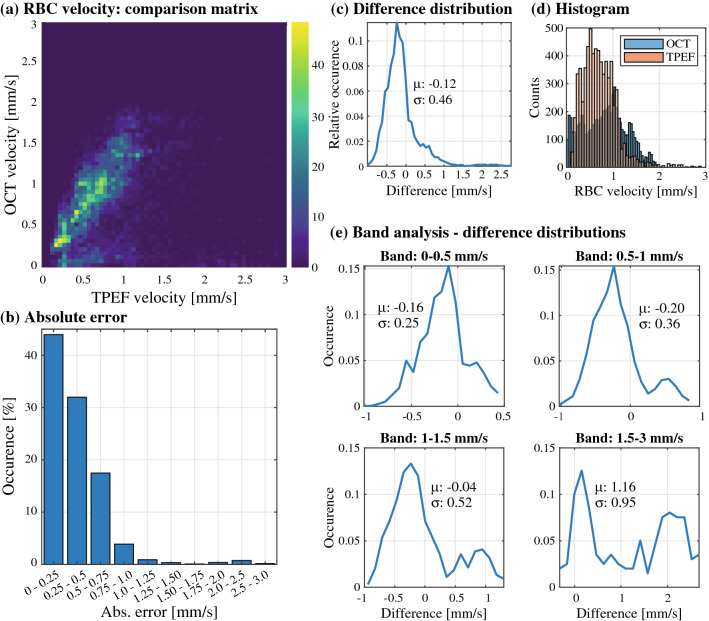
Evaluation of OCT RBC speed estimation. (**a**) 2D histogram displaying the estimation of RBC speed in both modalities, highlighting a concentration of measurements slightly above the diagonal, and a smaller cluster below. (**b**) Absolute error between both modalities, binned into steps of 0.25 mm/s. (**c**) Distribution of the differences in estimation for all measurements, with a mean of − 0.12 mm/s and variance of 0.46 mm/s. (**d**) Marginal histograms of OCT and TPEF RBC speed estimations, highlighting a more uniform distribution for OCT compared to TPEF. (**e**) Band analysis displaying the difference distributions for 4 bands of RBC speed: 0–0.5, 0.5–1, 1–1.5, 1.5–3 mm/s.

The results of the comparison are shown in Fig. 4. The 2D histogram shown in Fig. 4a shows an overall linear relationship between both modalities with a high variance (slope of 1.03, see Supp. Figure 4). A majority of the error lies within 0 and 0.75 mm/s, as highlighted in the absolute error distribution shown in Fig. 4b, and errors up to 3 mm/s were observed in the estimation. Overall, as displayed in Fig. 4c, the total difference distribution has a mean difference of − 0.12 mm/s and a variance of 0.47 mm/s, and is slightly skewed to negative differences (i.e. overestimation from the OCT metric, as the difference is defined as Δv = v_TPEF_ – v_OCT_). This observation is emphasized when comparing the individual velocity distributions of both modalities (Fig. 4d), as the OCT velocity estimates range up to 3 mm/s, whereas the TPEF measurements are mainly centered within the 0.2 and 1 mm/s range. Furthermore, the diagonal of the 2D histogram of Fig. 4a shows a rise in variance with increasing velocity. To investigate this observation, similarly to the RBC flux analysis, we computed the difference distributions of 4 velocity sub-bands (0–0.5 mm/s, 0.5–1 mm/s, 1–1.5 mm/s and 1.5–3 mm/s). As expected, these distributions, shown in Fig. 4e, show an increase in their variance for higher sub-bands. Interestingly, the OCT speed overestimation is primarily present for smaller velocities, as the distributions widen towards positive values with increasing TPEF speeds.

**Figure 5:**
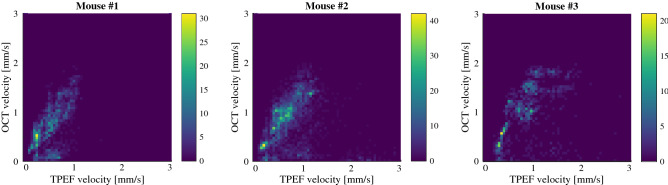
2D histograms highlighting the performance of RBC speed estimation between animals.

The trends described above can also be visualized in each isolated mouse, as is shown in Fig. 5. Although the individual 2D histograms seem to point towards an OCT overestimation for each mouse, a highly underestimated vessel in mouse #2 draws its individual linear fit towards a slope of 1 (see Supp. Figure 4). Additionally, for each mouse, two masses in the histograms can be observed, composed of either slightly overestimated (slightly above the histogram’s diagonal) or severely underestimated estimations (in the lower quadrants of the histogram).

## Discussion

### OCT estimates RBC flux best within 20–60 RBC/s

Our comparison between OCT estimates of RBC flux and ground truth measurements obtained in TPEF revealed a slight underestimation when considering every RBC/s bands. Nevertheless, the band analysis, shown in Fig. 2e, highlights the presence of a prominent bias for low and large RBC fluxes (0–20 RBC/s and 60–120 RBC/s respectively). In particular, for low RBC fluxes, although a large portion of the differences are concentrated around 0, the distribution spans to above 30 RBC/s. This overestimation tail originates from the misidentification of non-hemodynamic fluctuations in the OCT signal and the presence of double peaks on certain RBCs, as is shown with arrows on the time trace of Fig. 1d. Moreover, as OCT lacks specificity (as opposed to TPEF), the presence of additional non-hemodynamic fluctuations will hamper the RBC flux estimation. This is particularly problematic for low RBC fluxes, as a significant portion of the time traces are free of any RBCs but can comprise non-hemodynamic fluctuations (changes in position of vessel within mask, temporary increase in parenchyma’s scattering, decrease in scattering caused by overlying tissue etc.…). This problem seems to be less prominent for larger flows, as these additional fluctuations are overwhelmed by the frequent passage of RBCs.

For fluxes beyond 60 RBC/s, the primary cause of OCT underestimation might be the temporal sampling, as identified by Li et al^24^. In an attempt to alleviate this issue, we chose an interframe rate of 1.5 ms, which should theoretically allow us to measure beyond > 150 RBC/s. Nevertheless, as is shown in our analysis, bands beyond 60 RBC/s show an average difference > 6 RBC/s with a variance > 8.7 RBC/s (the highest variance of all bands). The first explanation for this divergence is the presence of speckle in OCT tomograms, which was not accounted for in the analysis performed by Li et al. Additional interference fringes caused by speckle might dampen the RBC peaks, or add weak fluctuations to the time trace making the RBC peak undetectable by the *findpeaks* function. The lateral resolution also plays a primary role in correctly identifying RBCs, especially for high RBC flux when RBCs are densely packed together. Although an in-depth analysis of the role of the lateral resolution is warranted, it appears particularly crucial for vessels with high linear density (RBC/mm), as the smoothing caused by the spatial filtering will attenuate the prominence of the RBC peaks, once again rendering them undetectable. It should be noted that the latter effect is observed for both OCT and TPEF timeseries.

Lastly, to assess the impact of the temporal sampling on the RBC flux performance, we numerically undersampled the datasets, as in Li et al^24^. By comparing the RBC flux obtained with undersampled OCT time traces and the TPEF ground truth, we obtain a similar trend as Li et al.^24^, as shown in Supp. Figure 5. Overall, undersampling reduces the RBC flux dynamic range, and a maximum RBC flux around 100 RBC/s, 80 RBC/s, 60 RBC/s, 50 RBC/s is obtained for a temporal sampling of 3 ms, 4.5 ms, 6 ms and 7.5 ms respectively. This temporal sampling analysis emphasizes the potential of faster OCT engines, such as novel swept-source systems with A-scan rates of a few MHz^25^, to further increase the dynamic range of RBC flux measurements.

### OCT RBC speed measurements are overestimated compared to TPEF

The velocity of RBCs was computed by converting the temporal spread RBC passages into speed estimates by assuming the RBC width. When compared to the ground truth, i.e. TPEF linescans, these OCT RBC speed estimates appear to be overall overestimated in average by 0.12 mm/s with a variance of 0.47 mm/s. The 2D histograms comparing the OCT and TPEF speeds (Fig. 4a) diverges slightly from its diagonal with increasing speeds, however a few severely underestimated vessels (forming a second cluster in the lower quadrant of the histogram) seem to reduce the overall average. This overestimation is observable when considering individual animals, as the linear fits of mouse #1 and #3 both have slopes > 1.1. Furthermore, the band analysis shows an increase in mean and variance for increasing velocities, and the presence of a second peak for velocities > 1 mm/s (which corresponds to the underestimated cluster in the 2D histogram).

Although a robust analysis is necessary to clearly underpin the cause of these two clusters, we speculate that the speed estimation error could origin both in a wrong assumption of the RBC width and the fitting of misidentified RBCs. As mentioned previously, the RBC’s speed is estimated through the ratio of its spatial width and its temporal spread as it crosses through the focus. Similarly to as in Lee et al.^21^, RBCs are approximated here as spherical scatterers with a fixed width of 6.5 µm. To assess the accuracy of this hypothesis, we isolated and performed a linear fit on the velocity estimations for each individual vessel (both in OCT and TPEF, fit forced to 0). We then adjusted the numerator of Eq. 2 in Lee et al.^21^ for each measurement by dividing the initial numerator (i.e. $num = \sqrt {w_{RBC}^{2} + w_{voxel}^{2} + w_{kernel}^{2} }$, with $w_{RBC} = 6.5$ µm, so $num = 7.38$ µm) by each individual slopes, essentially forcing each curve to 1. The new numerators obtained from this analysis are shown in Supp. Figure 6, revealing that the naïve width (i.e. $num = 7.38$ µm) overestimates the temporal spread of the passage of an RBC for a given speed, and a value around 5.13 µm would be more accurate. Using the same $w_{voxel}$ and $w_{kernel}$, we found that this new estimator of the numerator translates to an RBC width of $w_{RBC} = 3.75$ µm. This analysis might be approximative, as other factors might cause the overestimation observed in our evaluation, however it reinforces the notion that a fixed width of 6.5 µm might be overly conservative for this analysis, as it assumes that RBCs are rigid in capillaries, travel along their longest axis (flat faces parallel to orientation of vessel) and does not consider the vessel’s orientation. As the OCT RBC flux measurements do not rely on any assumptions regarding the RBC width, they are not affected by this potential source of error.

In addition to the potential inaccuracy caused by fixing the RBC’s width, another source of error could lie in the fitting of misidentified RBC peaks. Indeed, the velocity estimation hinges upon the assumption that each peak fitted corresponds to an actual RBC. However, as demonstrated earlier, the lowest and highest RBC flux bands (0–20 RBC/s, 60–120 RBC/s) over and underestimate the RBC flux respectively. In particular, for high RBC fluxes, the isolation of a single RBC peak for fitting becomes tedious, as densely packed RBCs produce close peaks with reduced amplitudes (the peaks do not go back to 0 before the arrival of another RBC). Additionally, high velocities intrinsically lead to less reliable fits, as narrower peaks are sampled by less timepoints. Ultimately, as vessels with higher RBC flux in our dataset typically have a higher average RBC speed, a combination of both issues mentioned above could explain the divergence observed in the velocity estimation. To shed light on this hypothesis, we split the velocity estimations in bands of RBC flux, as shown in Supp. Figure 7. The highest flux bands are overestimated and have the largest average differences (− 0.17 and − 0.16 for RBCs between 60–80 and 80–120 respectively). Interestingly, the average and variance of the distributions increase along with the RBC flux and reach a saturation point at around 40 RBC/s. Lastly, as will be discussed in the following paragraph, the angle of the vessel itself might be a source of error in the speed estimates.

### Limitations of the study

Our evaluation of the OCT RBC-passage technique is the first, to our knowledge, to compare hemodynamic parameters captured simultaneously from the same vessel with both OCT and TPEF. Combining both modalities required operating the TPEF microscope in slightly suboptimal conditions. Accordingly, as the OCT system comprises a moderately low numerical aperture (NA = 0.3), the TPEF excitation volume is larger than in optimal TPEF conditions (although recent wide-field TPEF systems operate with similar NAs^26,27^). Ultimately, this compromise limited our investigation to a depth of 100 µm below the pia, as the SNR of TPEF measurements dramatically decreased in deeper structures. Ultimately, a reduction in the SNR of the OCT signal with increasing depth could degrade the performance of the OCT RBC-passage technique investigated here. Furthermore, our analysis reflects the hemodynamic properties of shallow cortical depths: an average RBC flux and velocity of 50 RBC/s and 0.7 mm/s respectively. As deeper cortical layers have different hemodynamic properties, the technique’s performance could also diverge from the results obtained here^28,29^.

The angle of the vessel might also impact the speed estimates of both imaging modalities. Indeed, the speed estimates performed with TPM measure solely the velocity projection within the en face plane, and therefore do not capture the velocity component in the axial dimension. In OCT, the speed estimates would also depend on the vessel’s orientation if the PSF were anisotropic. In this case, the parameter $w_{voxel}$ should be adjusted to account for the capillary’s angle with the en face plane. In our analysis, the OCT resolution is almost isotropic, therefore we can assume that the vessel’s angle did not significantly impact the OCT speed estimations. Nevertheless, the TPM speed estimates are likely prone to underestimation, especially since the axial sectioning is relatively poor (due to the small NA). As the TPM speed estimates were treated as the reference, the angle dependency might partially explain the overestimation observed in the OCT speed estimations. A more detailed investigation underpinning the impact of the capillary’s angle on the speed estimates is warranted but lies outside of the scope of this manuscript.

Future work will thus focus on constructing a dual-modality system operating in ideal conditions for both TPEF and OCT. The analysis could have been undertaken using a high-NA objective (using an OCM system), however the overarching aim of this analysis was to evaluate the performance of the technique in typical OCT conditions.

## Conclusion

This work presents the first simultaneous and co-localized evaluation of an OCT metric for cerebral capillary imaging with TPEF microscopy, the gold-standard for cerebral vascular imaging. Through this analysis, we were first able to demonstrate that the passage of a RBC through the OCT’s focus causes a peak in the OCT intensity, as was suggested by Srinivasan et al.^14^, Ren et al.^22^, and Lee et al.^21^, amongst others. We also evaluated the accuracy of RBC flux and velocity measurements based on these fluctuations. We found that the RBC flux estimation with OCT is most accurate within the 20–60 RBC/s range, with an average difference between − 2.9 RBC/s and 0.9 RBC/s and a variance of 5.9 RBC/s. Below this range, the technique overestimates the RBC flux, whereas the opposite phenomenon is observed for larger RBC fluxes. The velocity comparison showed an overall difference of 0.12 mm/s with a variance of 0.47 mm/s. However, a significant portion of the OCT velocities were slightly overestimated compared to the TPEF measurements, which might be caused by assumptions on the RBC width and/or the fitting of misidentified RBC peaks. Altogether, despite the underwhelming performance of the technique in certain conditions (high speeds and high RBC fluxes), it should be emphasized that the RBC-passage method evaluated here remains amongst a handful of OCT methodologies enabling measuring hemodynamic parameters quantitatively in the cerebral cortex (i.e. RBC velocity and flux). Additionally, although a growing number of techniques are being developed to this aim, most have not been validated in vivo.

## Methods

### Multimodal imaging platform

A multimodal platform was designed for simultaneous OCT and TPEF in vivo imaging (shown in Fig. 1a and Supp. Figure 1). The TPEF microscope is composed of a femtosecond laser (MaiTai, Spectra-Physics), a Pockels cell (ConOptics) and PMTs (H7422, Hamamatsu Photonics, Japan). The detection and excitation paths are separated before the objective using a long-pass dichroic mirror. TPEF imaging was performed at 920 nm, and FITC fluorescence was collected through an emission filtered centered at 520 nm. Simultaneous TPEF and OCT imaging was performed using a 10 × 0.3 NA objective (Mitutoyo), providing a lateral resolution < 3 µm for TPEF imaging.

The OCT system consists of a light source operating at 1300 nm (LS2000C, Thorlabs), split into the sample and reference arm using a 90/10 fiber beam splitter, after crossing through a fiberized circulator. A commercial spectrometer was used to register interferograms, based on a high-speed 2048 pixels InGaAs line camera (Wasatch Photonics). Light from the OCT path was merged with the TPEF path using a short-pass dichroic mirror (Thorlabs) and then directed to the microscope’s objective. The OCT’s axial resolution, dictated by the source’s bandwidth is around ~ 5 µm in air (~ 3.8 µm in tissue), whereas its lateral resolution, given by the system’s effective numerical aperture, is ~ 3.5 µm. Dispersion was balanced by adding glass prisms (SF10 and UVFS) in the reference arm. Residual dispersion caused by the dichroic mirror was adjusted numerically.

A single computer controlled both OCT and TPEF platforms, but separate NI acquisition boards were used for each modality, as shown in Supp. Figure 1. Measurements with both imaging techniques was performed concurrently, but asynchronously (i.e. the OCT and TPEF imaging were started and stopped independently). Synchronization trigger signals enabled temporal alignment. For each measurement, the OCT imaging was initiated first, followed by the TPEF acquisition a few seconds later.

### Animal handling and training

Animal handling was performed in accordance with the ARRIVE guidelines and the recommendations of the Canadian Council on Animal Care. Surgical procedures and handling were approved by the ethics committee of the research center of the Montreal Heart Institute. Optical access to the cerebral cortex of c57bl/6 mice (n = 3, 3–6 months old, Charles River) was performed through a cranial window implantation, as previously described^30^. Briefly, animals were anaesthetized using isoflurane 1.5%–2.0% in pure oxygen). After exposing the skull, the exposed bone was covered with a layer of tissue adhesive and a titanium bar was fixed with dental cement. A circular region of 3 mm in diameter was drilled over the somatosensory cortex. The excised skull was then replaced by a stack composed of three 3 mm diameter coverglass and a 5 mm diameter coverglass, glued to the bone and sealed with dental cement. Care was taken not to touch or puncture the dura. The core temperature was maintained at 37 °C throughout the duration of the surgical procedure. One percent ketaprofen (SC, 5 mg/Kg, MERIAL Canada Inc) and 0.015 mg/mL buprenorphine (SC, 0.05 mg/Kg, Reckitt Benckiser Healthcare, UK) were administered before the procedure and during the two-day recovery period. After recovery, the mice were trained for awake imaging through head-fixing sessions of incrementally increasing time periods during 4 days (durations of 10, 20, 30 and 45 min respectively).

Prior to the imaging sessions, the blood plasma was labeled through a tail-vein injection of FITC (5%, 2MDa) for TPEF imaging. The mouse was then head fixed on a treadmill consisting of a 3D printed wheel, allowing free movement of the limbs while ensuring head fixation. The orientation of the mice’s head was slightly tilted before imaging, to place the glass window perpendicular to the optical axis. All imaging sessions were performed in the awake state.

### Measurement synchronization and co-localization

Synchronization of both imaging modalities was performed asynchronously, by recording TPEF signals on the OCT NI board. Two analog inputs on the OCT side recorded signals from the TPEF shutter and FITC PMT during the OCT acquisition at a rate of 10 kHz, to identify which OCT frames were acquired during TPEF imaging (Supp. Figure 1).

The spatial co-alignment between modalities was first performed coarsely on the microscope by adjusting some optical elements insuring both beams covered the same field-of-view. In a second step, a fine adjustment was performed by numerically correcting the OCT coordinates to match those of the TPEF channel. More specifically, a sample of Norland optical adhesive (NOA, Norland) was burnt at specific locations using the TPEF channel (by focusing the beam and increasing its power for a few seconds). This process changed the optical properties of the sample around the burnt area, which enabled visualizing the same structures in both TPEF and OCT channels. After imaging a grid of burnt spots with both imaging systems, the location of these spots in both channels was identified manually in post-processing, and transformation functions were established to convert coordinates chosen in TPEF into lateral positions for OCT imaging (Supp. Figure 2). This last step is crucial, as TPEF provides measurements covering a very restricted field-of-view, localized to only a single capillary vessel at a time.

### OCT acquisition protocol

After having selected a vessel of interest through the TPEF channel, the coordinates of the vessel were fed to the OCT acquisition interface. Before each OCT RBC-passage acquisition, a 2D angiogram in TPEF was obtained, covering the entire 500 × 500 µm^2^ field of view, as shown in Fig. 1b. The OCT signal fluctuations of the given vessel were then collected by acquiring repeated B-scans at 665 Hz (to obtain an interframe time of ~ 1.5 ms) along the system’s fast axis, around the vessel selected in the TPEF interface. The B-scan rate was chosen so as to minimize aliasing when registering peaks caused by the passage of RBCs and extend the theoretical RBC flux range to 100 RBC/s^24^. Each B-scan consisted of 60 × 2048 pixels, spanning 100 µm laterally, and each region was imaged for a duration of ~ 60 s (~ 40,000 B-scans per measurement).

An OCT angiogram^31^ covering the TPEF field-of-view was acquired at the beginning and end of each imaging session, as shown in Fig. 1c. Briefly, each lateral position was sampled twice along the slow-axis with interframe time of 10 ms, spanning a region of 500 µm × 500 µm laterally.

### OCT processing

Although the area covered by the B-scan (100 µm) is relatively small, the field-of-view still contained multiple capillaries. Before processing each measurement, an initial step of vessel identification was therefore necessary. To correctly identify the vessel, the vasculature in the OCT B-scans was first revealed through a point-wise complex subtraction^31^ and averaged over temporal dimension. The vessel imaged in TPEF was then identified in the aforementioned OCTA B-scan by comparing it with the 3D OCT angiogram and the 2D TPEF angiogram. Vessels which couldn’t be identified with certainty were discarded from the processing and the analysis. Additionally, vessels under large vessels with severe shadowing artefacts were removed from the analysis, as their fluctuations were contaminated by additional spurious hemodynamic activity.

With the vessel interrogated in TPEF identified, the OCTA B-scan was then used to create a mask around the vessel (to isolate solely the OCT signal coming from the vessel and not the parenchyma). The OCT intensity (magnitude of the OCT complex signal) was then isolated using the mask, high-pass filtered at 10 Hz to remove the DC component and then low-pass filtered with a gaussian support of 4 timepoints (σ_f_ = 1.8 ms). The time series were then split into sequences of 512 time points. For each sequence, a correlation analysis was performed between each time trace of each vessel pixel. From this correlation analysis, binary masks were obtained for each seed by thresholding the correlation degrees at 0.8. For each sequence, the masks were filtered using an image similarity metric, and the 4 largest and most frequent masks were selected. Finally, a similar mask filtering step was performed by comparing all of the masks for all of the sequences. The output of this analysis provided 4 different masks, the most frequent and largest masks obtained in the sequences. These masks provide areas within the vessel with highly correlated activity. This step is particularly important, as it allows averaging over areas of capillaries displaying similar hemodynamic activity. Depending on the orientation of the vessel, averaging over the initial mask (naïve surrounding of the vessel on the OCTA B-scan) might attenuate the prominence of peaks, through temporal wash-out, typically when the fast axis crosses the longitudinal axis of the vessel. This also allowed separating certain vessels imaged close to bifurcations. From these 4 masks, 4 individual time traces of the vessel’s activity were obtained by averaging the temporally filtered OCT intensity over the pixels in the different masks. Lastly, in a manual inspection, the time trace showing the least noise and most prominent hemodynamic activity (i.e. peaks caused by passage of RBCs) was selected within the 4 remaining time traces.

Prior to temporal alignment with the TPEF data, the trigger and PMT signals recorded with the analog input of the OCT NIDAQ board were resampled to match the B-scan acquisition frequency. Once resampled, the trigger and PMT signals were used to define the beginning and end of the OCT time trace (a threshold of 0.15 V was applied to define the beginning of the TPEF acquisition). A finer temporal alignment step was then performed when overlaying the OCT and TPEF time traces by identifying similar features between both traces (i.e. similar RBC peaks, stalling regions, motion artefacts…).

The location of peaks in the OCT time trace was performed similarly as Li et al.^32^, by using the *findpeaks* function in MATLAB. Prior to identifying the peaks, the OCT timeseries was once again split into sequences of 512 pixels, and each sequence was then normalized. Sequences containing several motion artefacts or stalls were excluded from the analysis. The identification of peaks in the OCT signal was then performed for each normalized sequence using the *findpeaks* function. The RBC flux (number of RBC per second) was then calculated by counting the number of peaks obtained per 665 timepoints (corresponding to one second of acquisition). With the peaks identified, their temporal width was then extracted by fitting each peak with a gaussian function. As described in Lee et al.^21^, the average RBC temporal width was obtained by averaging the variance estimates of each gaussian fits (for each peak of a given sequence). The speed was then calculated using the Eq. (2) in Lee et al., with *w*_RBC_ = 6.5 µm, *w*_voxel_ = 3.5 µm and *w*_kernel_ = 1.8.

All of the OCT angiograms obtained in this work were obtained through point-wise complex subtraction after motion artefact correction^33^.

### TPEF RBC flux and speed acquisition and processing

The ground truth for the hemodynamic activity of capillaries (RBC flux and speed) was obtained by acquiring so-called linescans over capillaries in TPEF^1^. Lines of 11.7 µm were acquired at a line rate of 800 Hz along the longitudinal axis of capillaries for a duration of approx. 60 s each per capillary. Prior to RBC extraction and speed computation, the TPEF linescans were downsampled to the OCT B-scan rate, i.e. down to 665 Hz. To extract time traces of RBCs crossing through the TPEF focus, a time series was extracted by selecting a lateral position over the linescan. To increase the SNR of the time series, a small region around the lateral position was selected, and the time traces were averaged after temporal realignment (to avoid any temporal washout). Similarly to the OCT RBC extraction, the timetrace was split into sequences of 512 timepoints and then normalized. The RBC peaks were then identified within the normalized sequences using the *findpeaks* function in MATLAB. The RBCs temporal locations were binned within steps of 1 s, exactly as performed with the OCT data (within sequences of 665 timepoints).

The 2D linescan data was also split into 2D sequences of 512 timepoints, and each the RBC velocity for each sequence was obtained through a Radon-based technique, based on the work in Drew et al^34^.

## Supplementary information

Supplementary information.
