# Supplementary material for: Validation of red blood cell flux and velocity estimations based on optical coherence tomography intensity fluctuations
Source: Sci Rep. 2020 Nov 11;10:19584. doi: 10.1038/s41598-020-76774-z (PMC7658245; doi:10.1038/s41598-020-76774-z)
Supplement: Supplementary file 1 — Supplementary information. [file 41598_2020_76774_MOESM1_ESM.pdf]

Validation of red blood cell flux and velocity estimations based on optical coherence tomography intensity fluctuations

Paul J. Marchand[1,2], Xuecong Lu[1,2], Cong Zhang[1,2] and Frédéric Lesage[1,2]

1. Department of Electrical Engineering, Polytechnique Montreal, Canada
2. Research Center, Montreal Heart Institute, Montreal, Canada

Supplementary Figures:

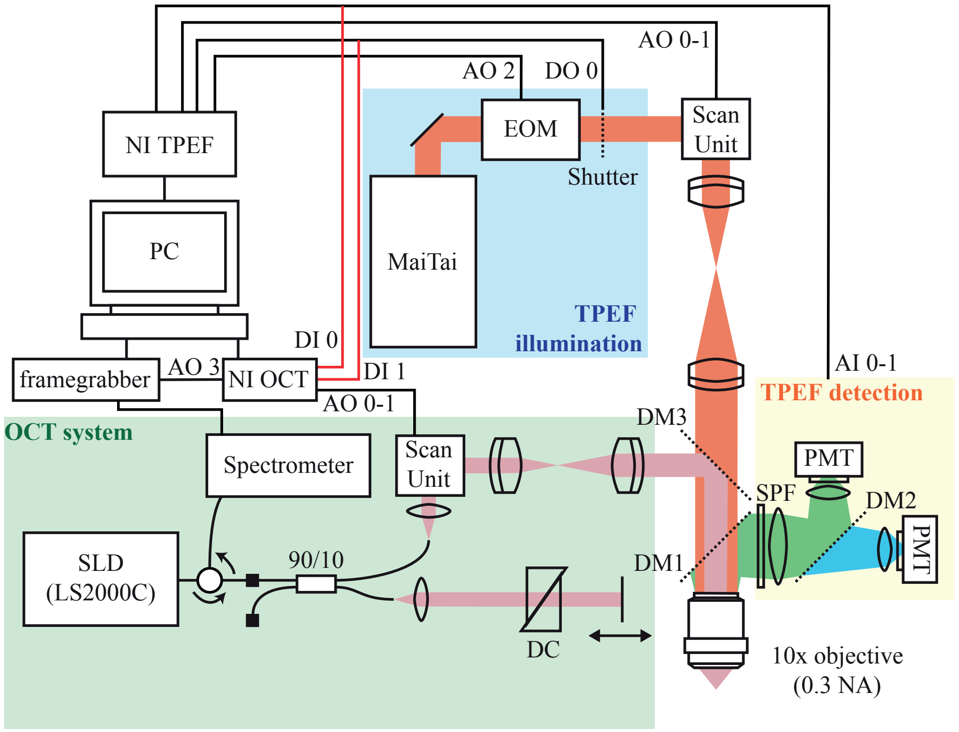

Supplementary Figure 1 *Complete schematic of the OCT/TPEF system used for the study. The OCT (in green) and TPEF (in blue) are controlled by the same computer through two individual NIDAQ boards. The TPEF NIDAQ controls the systems electro-optic modulator, the shutter and scan-unit; and registers the signals from the PMTs. The OCT NIDAQ controls the framegrabber and OCT scan-unit, and collects signals from the shutter and TPEF PMT for synchronization purpouses. The OCT is combined with the TPEF through a short-pass dichroic mirror before the objective. The TPEF illumination is collected through a long-pass dichroic mirror, and is then spectrally filtered and split into two different PMT channels. SLD: superluminescent diode, EOM: elecro-optic modulator, PMT: photomultiplying tube, DM: dichroic mirror, SPF: short-pass filter, DC: dispersion compensation, AO: analog output, AI: analog input, DI: digital input, DO: digital output, NI: national instrument DAQ board.*

Transformation between TPEF and OCT coordinates

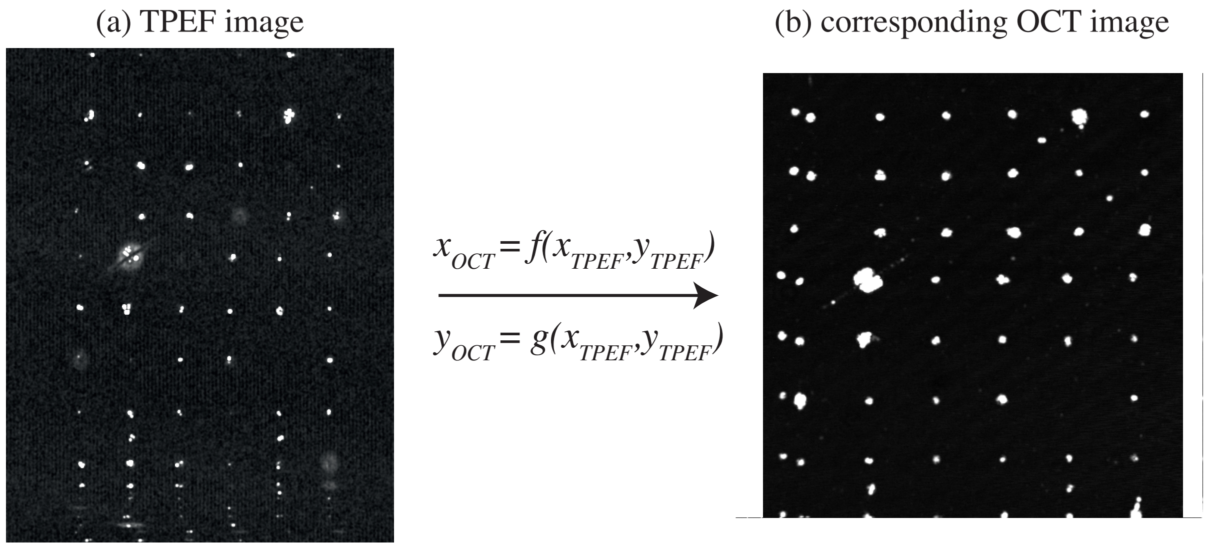

Supplementary Figure 2 *Transformation of TPEF coordinates into OCT coordinates. Structures burnt in Norland optical adhesive were imaged in both TPEF and OCT (a and b) and used to define transformation functions between both imaging systems.*

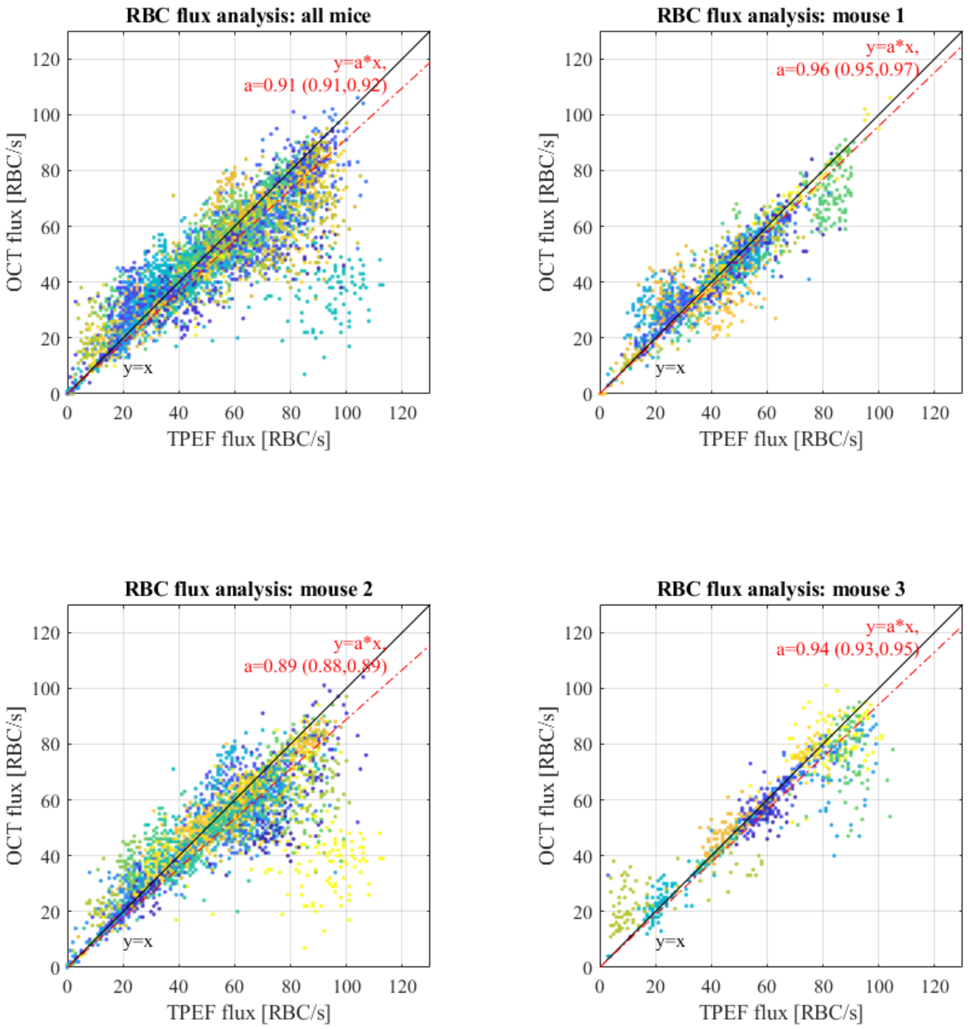

Supplementary Figure 3 *Scatter plots displaying the RBC flux estimations using TPEF and OCT, for the total dataset and the individual mice used in the study. Linear fits on the datasets highlight a consistent slight OCT underestimation, with a slope around 0.9. Mouse 2 has a lower slope of 0.89, mainly caused by a significantly underestimated vessel (yellow cluster). Each color encodes a distinct vessel.*

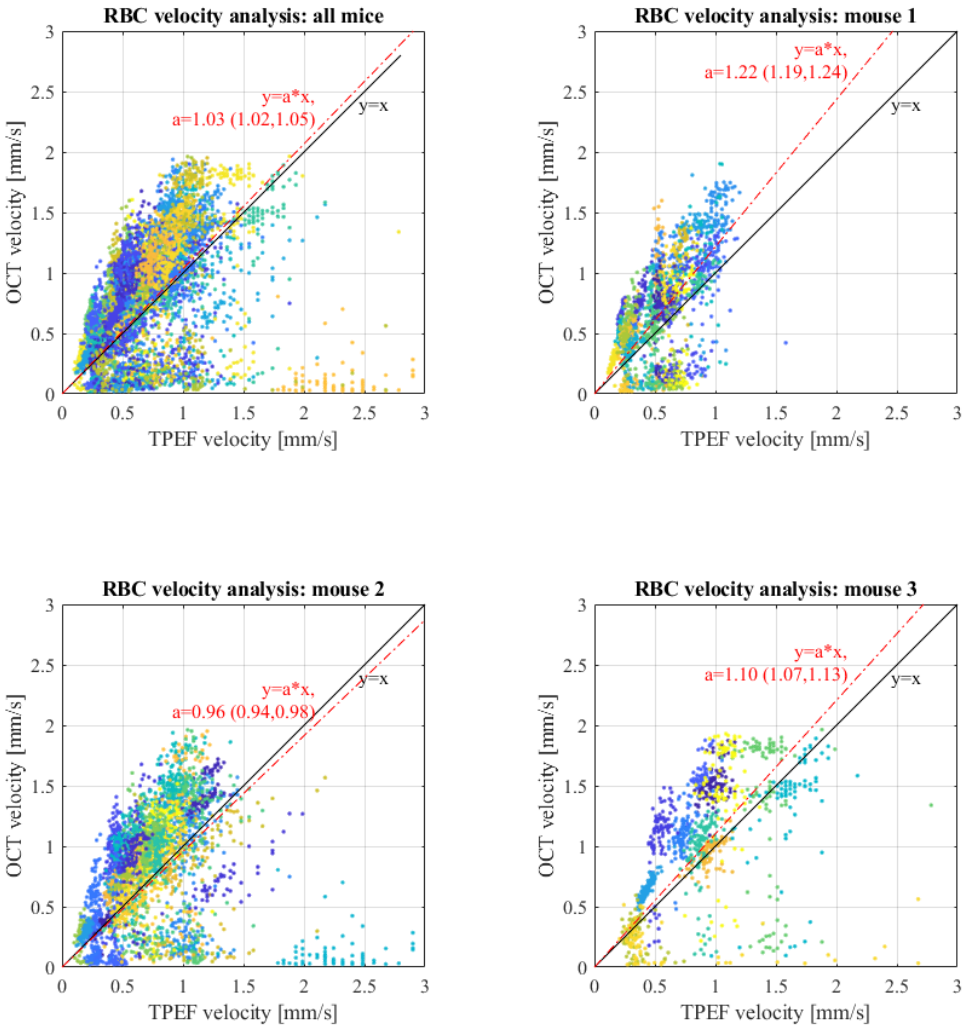

Supplementary Figure 4 *Scatter plots displaying the RBC speed estimations using TPEF and OCT, for the total dataset and the individual mice used in the study. Linear fits on the datasets highlight an OCT overestimation, with a slope above 1.1. Similarly to the RBC flux estimation, Mouse 2 has a lower slope of 0.96, mainly caused by a few significantly underestimated vessels (lower quadrant cluster). Each color encodes a distinct vessel.*

Sampling analysis:

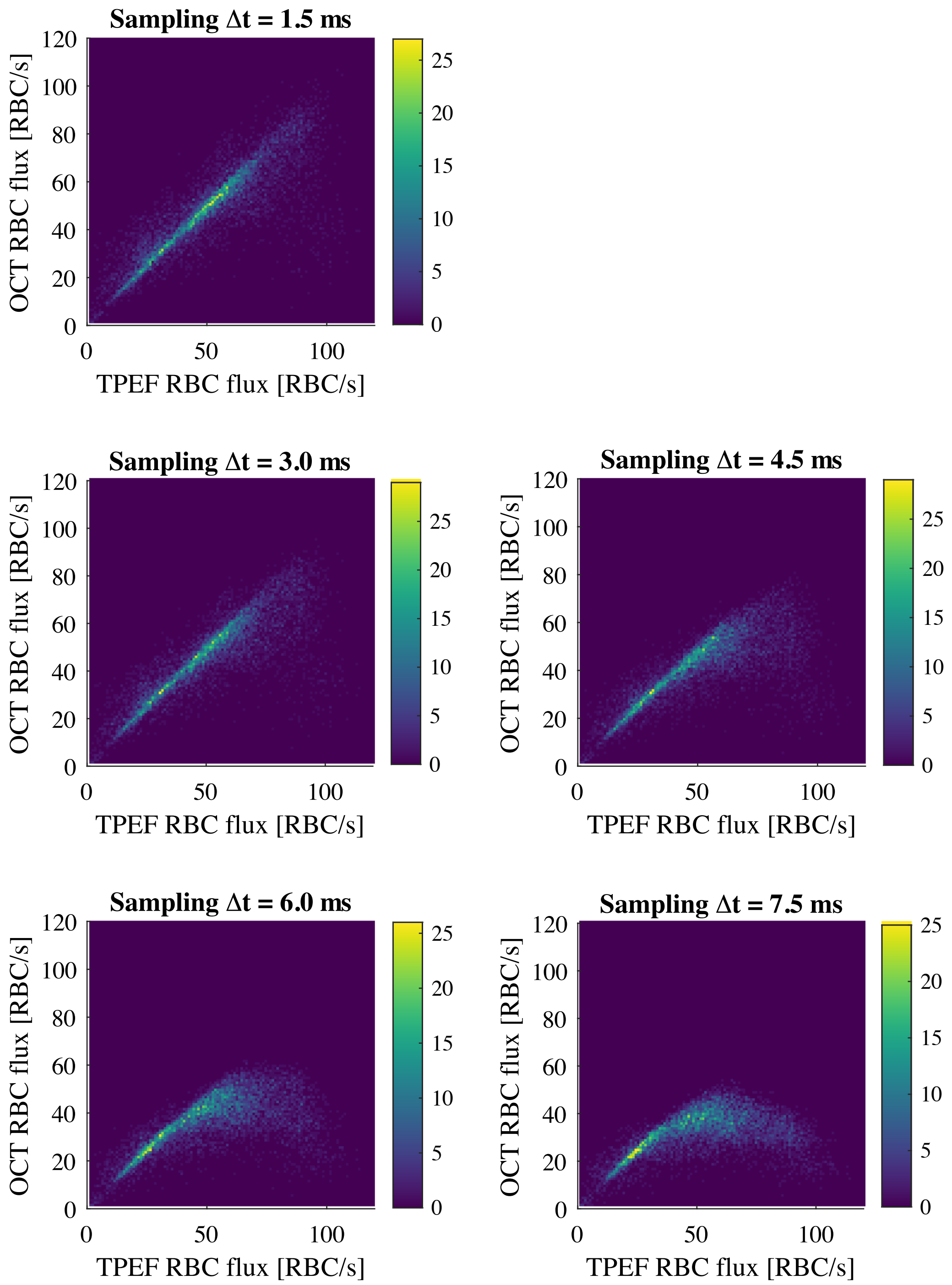

Supplementary Figure 5 *RBC flux validation for various temporal undersamplings. The timetraces obtained in OCT were undersampled to Δt = 3.0, 4.5, 6, 7.5 ms and used for RBC flux estimation. As expected, increasing the inter-Bscan period decreases the maximum RBC flux range, leading to a saturation around 40 RBC/s for 7.5 ms.*

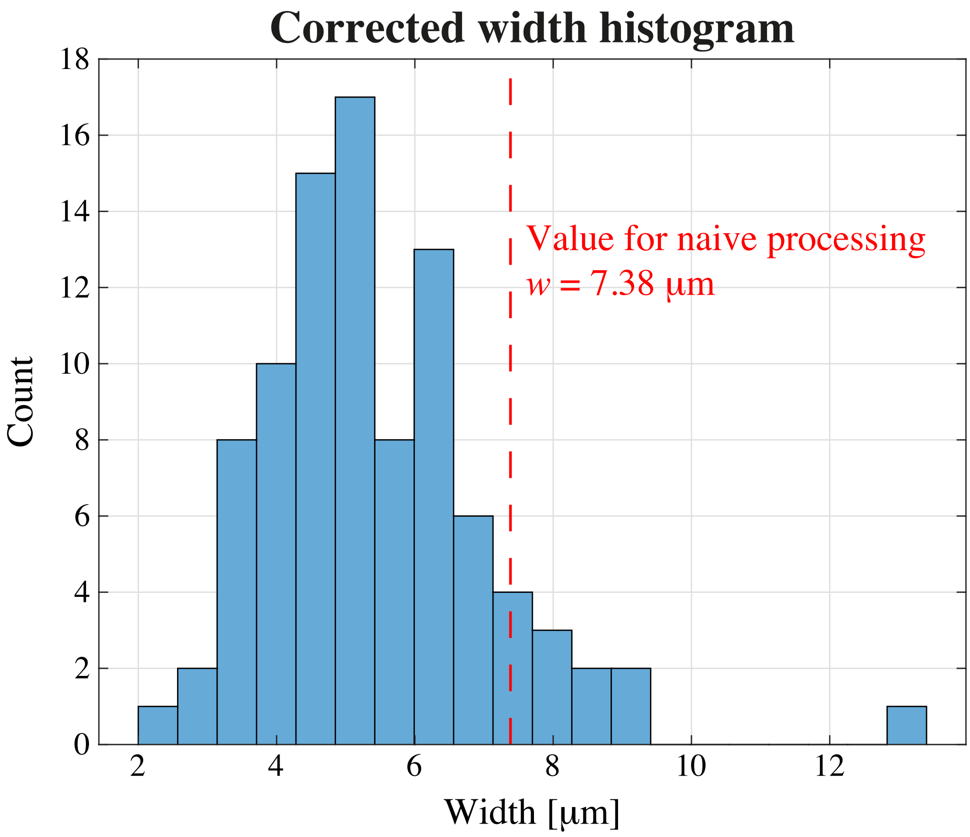

Supplementary Figure 6 *Distribution of the width necessary to obtain a slope of 1 for each speed estimation (for each vessel). The distribution points to an overestimation of the width used to compute the speed from the temporal spread of each passing RBC.*

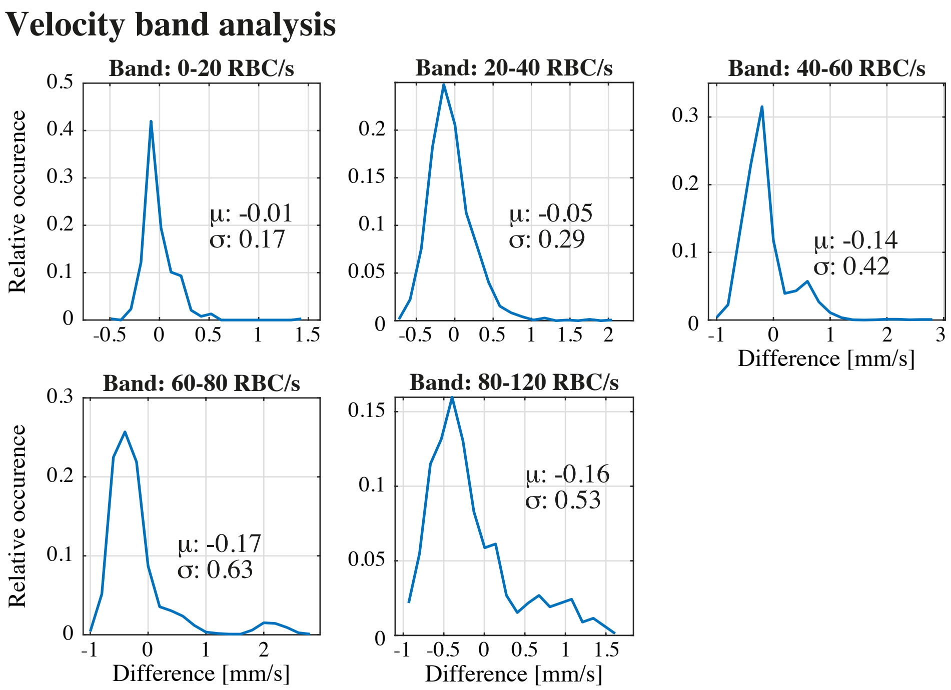

Supplementary Figure 7 *RBC flux Band analysis for the OCT RBC speed estimations. The RBC speed estimations were binned into RBC fluxes of 0-20, 20-40, 40-60, 60-80 and 80-120 RBC/s, and their difference distributions are plotted here. The analysis highlights that the speed estimations are in average more accurate for lower RBC fluxes. Interestingly, the mean and variance of the distributions reaches a plateau beyond 40 RBC/s.*
